# Persistent Fever in an Elderly Patient: Diagnostic Challenges and Management of Aspiration Pneumonitis and Organizing Pneumonia

**DOI:** 10.7759/cureus.70121

**Published:** 2024-09-24

**Authors:** Ryosuke Kashiwaba, Kohei Oka, Natsumi Yamamoto, Chiaki Sano, Ryuichi Ohta

**Affiliations:** 1 Family Medicine, International University of Health and Welfare Graduate School of Health Sciences, Tokyo, JPN; 2 Community Care, Unnan City Hospital, Unnan, JPN; 3 Community Medicine Management, Shimane University Faculty of Medicine, Izumo, JPN

**Keywords:** aspiration pneumonia, aspiration pneumonitis, corticosteroids, dysphagia, elderly patients, organizing pneumonia

## Abstract

This case report describes an 85-year-old woman with a history of aspiration pneumonia who was admitted to a rural hospital with fever, vomiting, and decreased oxygenation. Initially diagnosed with aspiration pneumonia and acute pyelonephritis, she was treated with antibiotics. Despite this, her fever persisted, and subsequent imaging suggested aspiration pneumonitis or organizing pneumonia. Her condition improved following fasting and corticosteroid therapy. This case highlights the challenges in differentiating aspiration pneumonia from aspiration pneumonitis, as both conditions may present similarly but require different treatment approaches. Persistent fever despite broad-spectrum antibiotics prompted a change in treatment strategy, leading to the introduction of corticosteroids, which improved her symptoms. This case underscores the importance of considering aspiration pneumonitis in older patients with recurrent or persistent respiratory symptoms and fever, especially when dysphagia is present and antibiotic therapy is ineffective. Early intervention with corticosteroids, particularly when imaging findings are suggestive of organizing pneumonia, can prevent further deterioration. Accurate diagnosis and timely treatment adjustments are crucial in managing aspiration-related pulmonary conditions in elderly patients.

## Introduction

The differential diagnosis of fever with respiratory symptoms in the elderly is often challenging due to the frequent presentation of nonspecific symptoms [1,2]. While pneumonia is the most common cause, distinguishing between aspiration pneumonia and aspiration pneumonitis leading to organizing pneumonia is essential, as their treatment strategies differ significantly [3,4]. We present the case of an 85-year-old woman with fever, vomiting, and decreased oxygenation, which underscores these diagnostic challenges. Initially diagnosed with aspiration pneumonia and acute pyelonephritis, she was treated with narrow-spectrum antibiotics. However, when this therapy failed, broad-spectrum antibiotics were introduced, but with no improvement [5]. This raised the suspicion of aspiration pneumonitis or diffuse aspiration bronchiolitis leading to organizing pneumonia [6]. Notably, her condition improved with fasting and prednisolone. Differentiation between these conditions typically requires a lung biopsy to detect the presence of aspirated food particles. However, in cases where biopsy is not feasible, clinical improvement with fasting and steroids may suggest aspiration pneumonitis with organizing pneumonia. This case highlights the importance of thorough clinical evaluation and an accurate diagnosis to ensure appropriate treatment.

## Case presentation

A care-dependent 85-year-old woman presented to a rural community hospital with fever, vomiting, and decreased oxygenation, all occurring one day before admission. Two months earlier, she had been treated at our hospital for aspiration pneumonia before being transferred to a care facility. She had no contact with patients with infections, including influenza, COVID-19, and tuberculosis. Her medical history includes hypertension, pyelonephritis, Alzheimer’s disease, iron deficiency anemia, atrophic gastritis, and previous episodes of aspiration pneumonia. Her current medications are donepezil 5 mg daily, candesartan 4 mg daily, amlodipine 5 mg daily, and lansoprazole 15 mg daily.

On admission, her vital signs were as follows: Japan Coma Scale score of 100, Glasgow Coma Scale score of 10 (E:2, V:2, M:6), blood pressure of 119/69 mmHg, heart rate of 127 beats per minute (regular), respiratory rate of 12 breaths per minute, oxygen saturation (SpO_2_) of 93% on six liters of oxygen, and a body temperature of 38.3°C. Physical examination revealed bilateral coarse crackles, a flat and soft abdomen without tenderness, and normal bowel sounds. No other neurological abnormalities were noted. Her skin was clear without eruptions.

Initial blood tests revealed elevated white blood cells, neutrophils, and C-reactive protein (CRP) levels, indicating a bacterial infection (Table 1).

**Table 1:** Initial laboratory data of the patient CRP: C-reactive protein

Marker	Level	Reference
White blood cells	2.5	3.5-9.1×10^3^/μL
Neutrophils	80.6	44.0-72.0%
Lymphocytes	15.3	18.0-59.0%
Hemoglobin	12.1	11.3-15.2 g/dL
Hematocrit	35.9	33.4-44.9%
Mean corpuscular volume	98.0	79.0-100.0 fL
Platelets	22.5	13.0-36.9×10^4^/μL
Total protein	6.9	6.5-8.3 g/dL
Albumin	3.7	3.8-5.3 g/dL
Total bilirubin	0.7	0.2-1.2 mg/dL
Aspartate aminotransferase	30	8-38 IU/L
Alanine aminotransferase	21	4-43 IU/L
Lactate dehydrogenase	237	121-245 U/L
Blood urea nitrogen	26.2	8-20 mg/dL
Creatinine	0.89	0.40-1.10 mg/dL
Serum Na	140	135-150 mEq/L
Serum K	4.6	3.5-5.3 mEq/L
Serum Cl	101	98-110 mEq/L
CRP	0.39	<0.30 mg/dL
Urine test	-	-
Leukocyte	Negative	Negative
Nitrite	Negative	Negative
Protein	Negative	Negative
Blood	Negative	Negative

**Figure 1:**
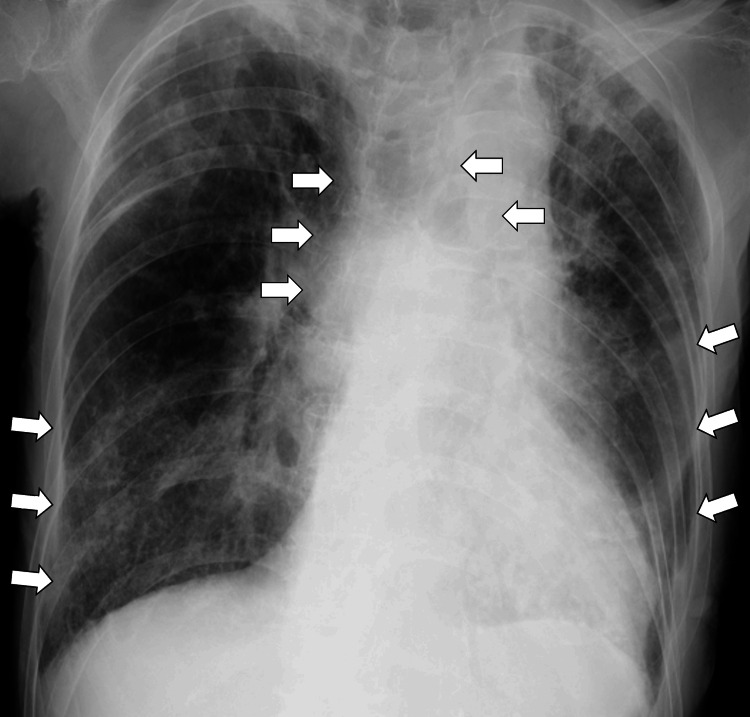
A chest X-ray showing decreased transparency in bilateral lungs and tracheal deviation to the left (white arrows)

Gram staining of the sputum showed a polymicrobial pattern, with predominant gram-negative rods and diplococci. A chest X-ray demonstrated decreased transparency and tracheal deviation to the left (Figure 1).

**Figure 2:**
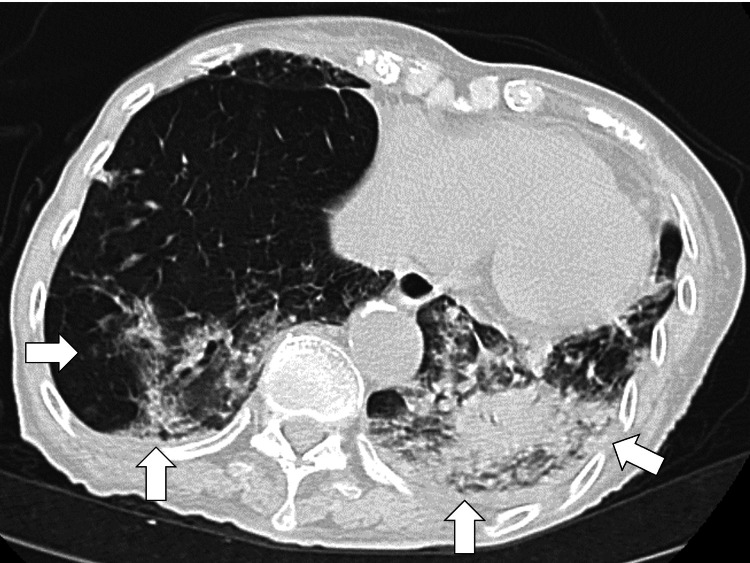
Chest CT revealing a scar-like lesion, granular shadows in the left lung, poorly defined infiltrates, and reduced lung transparency (white arrows) CT: computed tomography

Chest computed tomography (CT) revealed a scar-like lesion, granular shadows in the left lung, poorly defined infiltrates, and reduced lung transparency (Figure 2).

The patient was diagnosed with aspiration pneumonia due to vomiting and aspiration of oral bacteria. Initial treatment consisted of intravenous ceftriaxone (2 g per day) and seven-day fasting. By the second day, the patient became afebrile. On the third day, after confirming the safety of her swallowing function via videoendoscopic examination, oral intake was resumed. However, on the fourth day, she redeveloped a fever, reaching 38.5°C by evening, and a worsening cough. Sputum gram staining and culture revealed only normal oral flora, such as *Streptococcus dysgalactiae*. Given the concern for an extended-spectrum β-lactamase (ESBL)-producing organism, her antibiotic regimen was changed from ceftriaxone to cefmetazole (4 g per day).

**Figure 3:**
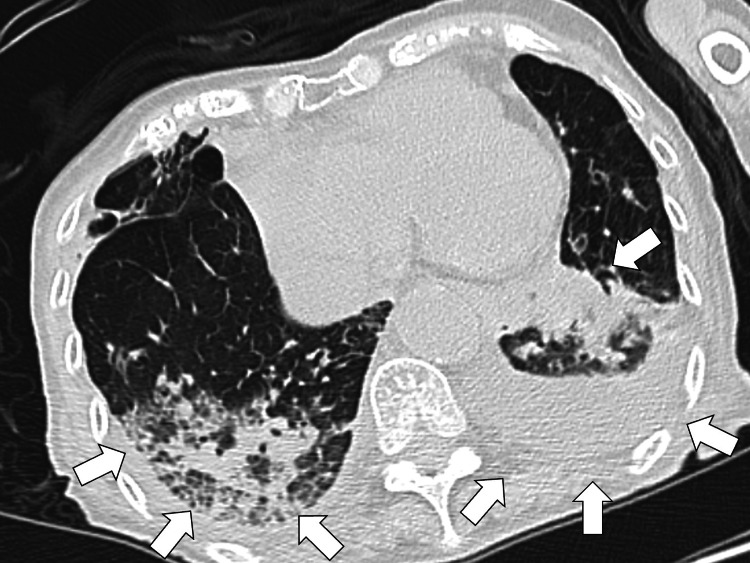
The chest CT revealing multiple infiltrates and ground-glass opacities in both lungs CT: computed tomography

Despite this change, her fever persisted. On the sixth day, oral intake was discontinued due to suspected asymptomatic aspiration, which reduced her fever to around 37°C. Although her cough improved, the mild fever persisted. On the 15th day of hospitalization, a chest-to-pelvic CT scan with contrast was performed to assess for abscess formation. The CT revealed multiple infiltrates and ground-glass opacities in both lungs, raising suspicion of aspiration pneumonitis leading to organizing pneumonia (Figure 3).

Prednisolone (30 mg daily) was initiated for one week and tapered at the pace of 5 mg per two weeks. By the 17th day, her fever had resolved. On the 20th day, based on swallowing video fluorography results, her diet was adjusted, and oral intake was resumed without recurrence of fever. The patient was then transferred to a rehabilitation unit in preparation for discharge.

## Discussion

This case report describes an older patient with persistent fever despite the use of broad-spectrum antibiotics, eventually diagnosed with aspiration pneumonia and organizing pneumonia. It highlights that assessing dysphagia in older patients using various tests may be inaccurate and that persistent aspiration can lead to ongoing fever and organizing pneumonia due to chronic inflammation [7,8]. General physicians should consider the possibility of occult aspiration in patients with persistent fever despite antibiotic therapy [9].

As this case demonstrates, pneumonia caused by aspiration can manifest as either aspiration pneumonia or aspiration pneumonitis, leading to organizing pneumonia. These two conditions often overlap in presentation, but their treatment strategies differ [10]. The treatment for aspiration pneumonia includes broad-spectrum antibiotics and swallowing rehabilitation [10]. In cases of aspiration pneumonitis, it is essential to assess improvement within 48 hours [3]. If no improvement is seen, treatment may involve broad-spectrum antibiotics, corticosteroids, and swallowing rehabilitation [10,11]. In this case, the initial assessment of the focus of fever tended to skew to infection, causing ineffective change of antibiotics and missed occult aspiration. Therefore, in elderly patients with multimorbidity, switching to broad-spectrum antibiotics should be carefully considered when initial treatment has failed.

Diffuse aspiration bronchiolitis and pneumonitis are related to aspiration pneumonitis with organizing pneumonia. Treatment for diffuse aspiration bronchiolitis and pneumonitis includes broad-spectrum antibiotics, bronchodilators, corticosteroids, and swallowing rehabilitation [12]. Recurrent pneumonia is a hallmark of this condition, and preventing further aspiration is critical to management [13]. In this case, despite broad-spectrum antibiotics, there was no improvement [14]. After starting corticosteroids, the patient’s symptoms were alleviated and cured. This suggests that early corticosteroid use, suspecting the complication of organizing pneumonia, should be considered when no infectious signs are identified in patients with aspiration pneumonitis and persistent fever. 

Chest CT in aspiration pneumonia and pneumonitis cases can reveal micronodules and tree-in-bud opacities, findings associated with mycobacterial, fungal, or bacterial infections, and tuberculosis [15]. Ruling out infection before starting steroids is mandatory for avoiding the worsening of occult infection [16]. To rule out the possibility of infection, taking a past medical history of exposure to tuberculosis and transient treatment of antibiotics for pneumonia are reasonable [17]. Our treatment strategy aligned with prior studies, escalating to broad-spectrum antibiotics and implementing fasting [17]. However, since symptoms did not improve, corticosteroids were initiated. Although made before a definitive diagnosis, this early intervention was crucial in preventing disease progression. General physicians are demanded to approach various cases of older patients suspecting aspiration pneumonia. Thus, consideration of the coexistence of aspiration pneumonia and pneumonitis triggering organizing pneumonia is essential, and comprehensive approaches through interprofessional collaboration assessing dysphagia are needed [18,19].

## Conclusions

An elderly woman with respiratory symptoms and fever was initially treated with antibiotics for suspected pyelonephritis and aspiration pneumonia, but her condition deteriorated. Subsequent imaging suggested aspiration pneumonitis or diffuse aspiration bronchiolitis, which improved with fasting and corticosteroid therapy. Accurate differentiation between aspiration pneumonia and pneumonitis is critical, mainly when antibiotics fail, as treatment approaches differ significantly. While identifying food debris via lung biopsy can aid diagnosis, clinical response to treatment may guide diagnosis in cases where biopsy is not feasible.
